# Paramount therapy for young and fit patients with mantle cell lymphoma: strategies for front-line therapy

**DOI:** 10.1186/s13046-018-0800-9

**Published:** 2018-07-13

**Authors:** Haige Ye, Aakash Desai, Shengjian Huang, Dayoung Jung, Richard Champlin, Dongfeng Zeng, Fangfang Yan, Krystle Nomie, Jorge Romaguera, Makhdum Ahmed, Michael L. Wang

**Affiliations:** 1 Department of Hematology, The First Affiliated Hospital of Wenzhou Medical University, Wenzhou, China; 2 Department of Lymphoma/Myeloma, The University of Texas MD Anderson Cancer Center, 1515 Holcombe Blvd, Houston, TX 77030 USA; 3 University of Texas Health Science Centre at Houston, Houston, TX USA; 4 Division of Cancer Medicine, Department of Stem Cell Transplantation and Cellular Therapy, The University of Texas MD Anderson Cancer Center, Houston, TX USA

**Keywords:** Mantle cell lymphoma, Young fit, Intensive therapy, Front-line

## Abstract

The natural history of mantle cell lymphoma (MCL) is a continuous process with the vicious cycle of remission and recurrence. Because MCL cells are most vulnerable before their exposure to therapeutic agents, front-line therapy could eliminate MCL cells at the first strike, reduce the chance for secondary resistance, and cause long-term remissions. If optimized, it could become an alternative to cure MCL. The key is the intensity of front-line therapy. Both the Nordic 2 and the MD Anderson Cancer Center HCVAD trials, with follow-up times greater than 10 years, achieved long-term survivals exceeding 10 years. But the Achilles heel in both trials were the severe toxicities, such as secondary malignancies including myelodysplastic syndromes /leukemia. Therefore, intensive therapies can act as a double-edged sword providing long term survival at the cost of severe toxicities. In our opinion, although intensive chemotherapy can cause detrimental side effects, it is indispensable given that we run the risk of sacrificing long-term survivals in these young and fit patients. We must seek for a powerful alternative at the front-line. Furthermore, minimal residual disease negativity should be the optimal therapeutic goal to achieve before and after autologous stem cell transplantation. Some novel therapeutic strategies have shown to improve outcomes, but it is not yet clear as to how these results translate in population. Of note, MCL patients need to be stratified at diagnosis and be provided with different intensities of front-line regimen. In this review, we discuss current strategies for the treatment of young patients with newly diagnosed MCL.

## Background

Mantle cell lymphoma (MCL) is an aggressive and incurable subtype of B cell lymphoma. The natural history of MCL is characterized by progression from remission to recurrence, which eventually leads to death from the disease due to complete resistance. Tumor cells prior to being exposed to therapeutic agents are most vulnerable to front-line therapy and acquiring drug resistance. An ideal front-line therapy could eliminate all tumor cells at first strike, reducing the chance of secondary resistance and causing long-term remissions that will eventually lead to a complete cure. Thus, the quality and intensity of front-line treatments are critical as they may become an alternative to cure MCL [1–4].

The median age at onset of MCL is 65 years and about a half of the population is younger than 65 years [5, 6]. However, there is a limited number of phase II, phase III and randomized clinical trials on these young MCL patients, with no standard treatment for the newly diagnosed cases. The current trend in treatment of young MCL patients is based on the results of phase I and II clinical trial data, which vary considerably among different centers. Since MCL is a rare tumor, numerous phase III trials are delayed and also difficult to perform. Therefore, MCL therapy has been largely based on phase II trials and only a few phase III trials.

Currently, the best survival data for young and fit patients (i.e. ≤ 65 years) with MCL is from a phase II clinical trial conducted at MD Anderson Cancer Center (MDACC), which reported a median overall survival (mOS) of 10.7 years with a median follow up of 13.4 years [7]. In addition, the Nordic trial reported a projected 10-year OS of 58%, with a median follow up of 6.5 years [8]. The 15-year updated results of the Nordic MCL2 study after a median follow-up of 11.4 years showed 12.7 years of OS and a 40% remission after 12 years [9].

Encouraging results have been obtained by a series of phase II studies including high-dose therapy followed by autologous stem cell transplantation (ASCT), which could eliminate residual lymphoma cells after conventional chemotherapy [10–18]. For those who are young, fit, and receiving less intensive chemotherapy as a front-line therapy, such as the R-CHOP (rituximab, cyclophosphamide, doxorubicin, vincristine and prednisone) regimen, survival was not significantly prolonged with an at best median survival of 5 years [19, 20]. Subsequently, the European MCL Network compared ASCT to interferon-α maintenance after CHOP-like induction and demonstrated a prolonged progression-free survival (PFS), although the comparison of OS still needs to be determined after a longer follow-up [21]. In this review, we focus on the various treatment strategies available for treatment of MCL in young, fit individuals and assess the efficacy of each treatment option to determine the paramount therapy for MCL in this age group.

### Treatments

Since many factors influence MCL prognosis, therapies should be tailored to achieve maximum effectiveness with minimal toxicity. The best chance for success would be at the time of diagnosis, when the malignant cells have not yet been exposed to any treatment drugs and are most vulnerable to therapy.

Different treatments have varying intensity. For example, conventional R-CHOP and BR (bendamustine plus rituximab) regimens can be viewed as a “less intensive therapy.” However, these treatments show at best a survival of only 5 years and clearly do not have strong enough evidence to be used as front line therapies. An improvement in OS has been reported with the use of more intensive therapies, such as R-high-dose cytarabine (HD-Ara-c) chemotherapy followed by ASCT and R-Hyper-CVAD (fractionated cyclophosphamide, vincristine, doxorubicin, and dexamethasone) alternating with R-methotrexate-cytarabine (MA) without high dose chemotherapy/ autologous stem cell rescue (HDT/ASCR) as consolidation, which can improve the median survival to more than 13 years (Table 1) [7, 21–25]. Still, the acute and long-term toxicities caused by these intensive treatments, including secondary malignancies, are a cause of concern. Therefore, treatment individualization based on a validated prognostic tool is crucial.

**Table 1:** Intensive front-line therapy for young fit patient with mantle cell lymphoma (MCL)

Regimen	Series	No.	Age(ys) Median (range)	MIPI Low/Int/High(%)	ORR (%)	CR (%)	mFU (ms)	mPFS (ms)	mOS (ms)	TRM (%)	Second malignancy(%)
R-hyper-CVAD/R-MA	Chihara (2016) [7, 27]	65	56(41–65)	5.52(4.87–6.91) Score	97 (of 97 cases)	89	13.4 ys	6.5 ys	13.4 ys	6.5	26
R-hyper-CVAD/R-MA	Merli (2012) [24]	60	57(29–66)	60/31/9	83	72	46	61% (5-y PFS)	73% (5-y OS)	5	1.6
CHOP-Like + interferon-α VS. CHOP-Like + ASCT	Dreyling(2005) [21]	60	55(37–65)	42/54/4 (IPI)	99 Vs 98	37 Vs. 81	34	17	3-y OS, 77%	0	na
		62	56(35–65)	44/49/7(IPI)				39	3-y OS, 83%	5	na
R-CHOP+ASCT Vs. R-CHOP/R-DHAP + HD-Ara-c + ASCT	Hermine (2012) [31, 32]	234	55 (48–60)	60/26/14	97. Vs 97	63 Vs. 65	6.1ys	3.9 ys^a^	5-y OS, 40%	3.4	5.2
		232	56 (50–60)	65/22/13				9.1 ys^a^	5-y OS, 65%	3.4	6.7
R-CHOP + MTX + R + HD-AraC/etoposide + ASCT	Damon (2009) [63]	78	57 (37–69)	53/31/15 (1 Unknown)	88	69	4.7ys	56% (5-y PFS)	64% (5-y OS)	2.6	na
R-CHOP + HD-Ara-c + ASCT	Van’t Veer (2008) [64]	87	55 (32–66)	51/29/20	70	64	41.7	44% (4-y PFS)	66% (4-y OS)	6.9	na
R-CHOP/R-DHAP + HD-Ara-c + ASCT	Delarue,2013 [33]	60	57 (40–66)	55/32/13	82	78	5.6ys	64% (5-y EFS)	75% (5-y OS)	1.7	18
R-DHAP + ASCT	Le Gouill (2014) [65]	299	57 (27–65)	53.2/27.4/19.4	na	92	35.8	74% (3-y PFS)	83% (3-y OS)	na	na
R-CHOP+R-CTX + R-HD-Ara-c + ASCT +/− LEN	Cortelazzo(2015) [54]	266	57 (51–61)	Int-High:53	86	77	19	77% (2-y PFS)	88% (2-y OS)	1.6	na
R-maxi-CHOP^b^/HD-Ara-c + ASCT +/− R	Eskelund (2016) [9]	159	56 (32–65)	51/26/23	96	89.7	11.4ys	8.5ys	12.7ys	7	12.6
R-CHOP-14 + (R)ICE +ASCT	Schaffel (2010) [66]	69	< 60, 71% ≥60, 29%	19/49/32	na	na	4.8ys	65% (4-y PFS)	84% (4-y OS)	1	na

*Abbreviations: HD-Ara-c* high-dose cytarabine, *ASCT* autologous stem-cell transplantation, *R* rituximab, *CR* complete response, *MIPI* mantle cell lymphoma-international prognostic index, *Int* intermediate, *mFU* Median follow-up, *mPFS* median progression-free survival, *EFS* event-free survival, *mOS* median overall survival, *ORR* overall response rate, *TRM* treatment-related mortality; ^a^ *TTF* time to treatment failure

*NR* not reached, *na* not available, *ne* not evaluable, *ms* months, *ys* years, *vs.* versus

hyper-CVAD/MA, fractionated cyclophosphamide, vincristine, doxorubicin, and dexamethasone alternating with methotrexate-cytarabine;

DHAP, dexamethasone, cytarabine, carboplatin or cisplatin; ^b^maxi-CHOP: cyclophosphamide 1200 mg/m ^2^, doxorubicin 75 mg/m ^2^, vincristine 2 mg total day1;prednisone 100 mg days1–5; LEN: lenalidomide; ICE, ifosfamide, carboplatin, and etoposide

Even in younger patients, there are a small number of patients with indolent tumor characteristics who are classified as low risk according to the Mantle Cell International Prognostic Index (MIPI) and/or Ki-67 proliferation index. The need for an intensive therapy for these cases can be uncertain, usually creating a dilemma to the clinicians. Below we discuss various treatments used as the front-line therapy for young and fit patients with MCL, which may better provide clinicians with an appropriate strategy in therapy selection. Furthermore, it may help clinicians to design their own clinical trials based on existing evidence.

**Intensive therapy** (Table 1)**:**

### Intensive therapeutic regimens

A prospective multicenter study reported by LaCasce et al. from the NHL database of National Comprehensive Cancer Network (NCCN) compared RCHOP with other intensive therapies in 167 young untreated MCL patients (median age: 56 years, range: 29–64). Intensive therapies included RHyper-CVAD, RCHOP + HDT/ASCR and RHyper-CVAD + HDT/ASCR. After 33 months of median follow-up, the median PFS in intensive therapy groups was significantly longer compared to the RCHOP group (3-year PFS, RHCVAD group: 58%; RCHOP + HDT / ASCR group: 56%; RHCVAD + HDT / ASCR group: 55%; RCHOP group: 18%, *p* < 0.004, *p* < 0.001, *p* < 0.001), although there was no statistically significant difference in the OS between the two groups. This could be attributed to the small sample size and short follow-up time (only 2–3 years). Additionally, mPFS of these intensive groups was still only 3–4 years, which was not satisfactory [26]. The following includes three regimens that are considered to be intensive: Hyper-CVAD/MA, HD-Ara-c followed by ASCT, and R-DHAP followed by ASCT.

#### Hyper-CVAD/MA regimen

Romaguera et al. reported a Phase II study of hyper-CVAD/MA regimen at MDACC, which included 65 younger patients (≤ 65 years old) with newly diagnosed MCL in a total of 97 cases. A recent 15-year follow-up report of this regimen (median follow-up 13.4 years) shows a mOS of 13.4 years for younger patients, with no recurrence in nearly one-third of the patients. This regimen also achieved a complete response rate (CR) of 89% in younger patients [27]. However, the toxicity treatment-related mortality (TRM) was 6.5% in patients less than 60 years old including 2 infections and 1 from an unknown cause. Twenty-five patients (26.0%) experienced secondary malignancies. The secondary malignancies were myelodysplastic syndrome/acute myeloid leukemia (MDS/AML, *N* = 13), prostate cancer (*N* = 4), lung cancer (*N* = 2), esophageal cancer (*N* = 1), renal cell carcinoma (*N* = 3), marginal zone lymphoma (*N* = 1), Burkitt lymphoma (*N* = 1), multiple myeloma (*N* = 1), pancreatic cancer (*N* = 1), colon cancer (*N* = 1) and thyroid cancer (*N* = 1). Six patients experienced MDS/AML in first remission while three patients experienced multiple malignancies. The incidence of a 10-year cumulative MDS/AML and all type of secondary malignancy for young patients in remission following R-HCVAD/MA was 6.2% (95% CI: 2.0–13.8%) and 15.4% (95% CI: 7.9–25.1%), respectively [7].

Based on the data from a single center trial at MDACC, clinical effectiveness and feasibility for RHyper-CVAD / RMA regimen requires more multicentered clinical trials. From 2005 to 2010, a prospective multicenter study from Gruppo Italiano Studio Linfomi (GISL) tested this regimen in 60 patients with a median age of 57 years old (29–66). They received 4 courses of R- hyper-CVAD/MA. If CR was not achieved after induction chemotherapy, ASCT followed. The RHyper-CVAD/ RMA regimen showed a good overall response rate (ORR) (83%) and CR (72%). At the median follow-up of 46 months, the estimated 5-year OS and PFS were 73 and 61%, respectively. The prognosis of low-risk MIPI score patients (estimated 5-year OS was 89 and 80%) was significantly better than the high-risk patients (24%). As expected, toxicity was evident, with 5% of TRM and 2% incidence of secondary AML [24]. However, a higher rate of treatment failure was observed in comparison to the MDACC trial. Only 37% of the patients (22 cases) completed the four courses of treatment in this study and 61% of patients completed the planned course of treatment in the SWOG multicenter, while the rate was 70% for the MDACC study [25].

Furthermore, a multicenter phase II study from SWOG showed that RHyper-CVAD/RMA was an effective regimen. The SWOG 0213 study treated 49 cases of newly diagnosed MCL patients with progressive stage with a median age of 57.4 years (35–69.8). The results also showed good ORR (86%). However, in comparison to the MDACC GISL trial, they had a significantly lower CR rate (47%). After a median follow-up of 4.8 years, the mPFS was 4.8 years (5.5 years for those ≤65 years) and mOS was 6.8 years. The low to intermediate-risk MIPI score accounted for 86% of patients. TRM for one and secondary MDS for two patients were observed [25]. Recently, another clinical trial from SWOG regarding RHyper-CVAD/RMA versus R-bendamustine followed by ASCT has been terminated due to insufficient collection of stem cells in the Hyper-CVAD/MA group. To improve the shortcomings of this regimen, which includes treatment-related complications (i.e. neutropenia), more intensive therapies are needed.

To summarize, the R-hyperVCAD/MTX/cytarabine regimen indeed demonstrates remarkable efficacy for MCL patients ≤65 years of age in the trials performed in MDACC, SWOG and GISL. Despite of the difference in patient characteristics in these three studies, considerably high ORR (> 80%) and CR/CRu (> 70%) were achieved in younger patients except for in the SWOG study, which had a CR/CRu of only 55%. Furthermore, the estimated 5-year OS and PFS were beyond 50% for younger patients. Meanwhile, encouraging results were shown by MDACC with the longest median survival of 13.4 years due to the longest median follow-up time of 13.4 years. Of note, the fact that at least half of the patients in these three studies were at low risk by MIPI indicates low risk patients may benefit most from this intensive treatment. Alternatively, the overall outcome may be driven in part by the favorable outcome of this low-risk group. However, toxicity was also very obvious, with incompletion of the planned courses for 29% in MDACC, 39% in SWOG, and 63% in GISL. The higher rate of incompletion of courses in GISL was possibly influenced by more patients being aged > 60 years (35%). Thus, this regimen may be too toxic in community hospital settings.

#### HD-Ara-c as a single agent followed by ASCT (Nordic trial) (Table 2)

The European Group for Blood and Marrow Transplantation (EMBT) and the European Mantle Cell Lymphoma Network (EMCL) recommends that the induction therapy for MCL to include HD-Ara-c plus rituximab [28].

**Table 2:** Nordic treatment regimen for MCL

	MCL1: 1996–2000 [16]	MCL2: 2000–2006 [8, 9]	MCL3: 2005–2009 [29]
No.	41	160	160
Median age (range), y	56 (38–65)	56 (32–65)	58 (28–65)
MIPI score (%) Low/Int/High	8/46/23/23^a^	51/26/23	48/31/21
Induction	Maxi-CHOP21^a^	R-Maxi-CHOP21 alternating with R-HD-Ara-c	R-Maxi-CHOP21 alternating with R-HD-Ara-c
Intensification therapy	BEAM/BEAC+ASCT	BEAM/BEAC+ASCT+/− rituximab	BEAM/BEAC+ (Zevalin, if<CR) + ASCT
ORR(%)	96	96	97
CR (%)	89	54	82
Follow-up (ys)	2.8	11.4	4.4
mPFS (ys)	4-y FFS, 20%	8.5	NR (4-y PFS, 71%)
mOS (ys)	4-y OS, 61%	12.7	NR (4-y OS, 78%)
TRM (%)	2	7	6
Second malignancy(%)	7	6	4

Abbreviations as indicated in the Table 1

Note: *FFS* failure-free survival, ^a^ *IPI* Low/Low-Inter/Inter-High/High; *BEAM/BEAC *Carmustine, etoposide, cytarabine, and melphalan or the same regimen with cyclophosphamide instead of melphalan; Zevalin, ^90^Y-ibritumomab-tiuxetan

The addition of HD-Ara-c as a single agent to intensive therapy followed by supportive stem cell regimen was studied by the Nordic group in a non-randomized phase II multicenter trial [22]. The Nordic Lymphoma Group (NLG) conducted its first MCL phase II protocol (NLG MCL-1) in 1996 to 2000 with an induction treatment of 4 cycles of dose-intensified CHOP without rituximab, followed by carmustine, etoposide, cytarabine, and melphalan (BEAM) or the same regimen with cyclophosphamide instead of melphalan (BEAC), high-dose chemotherapy with unpurged or ex vivo–purged ASCT. Forty-one newly diagnosed patients below 66 years were enrolled and given three series of an augmented CHOP regimen. Responders underwent stem cell mobilization with a fourth course of CHOP, stem cell harvest, and ASCT. However, the results were disappointing. 85% of the patients failed the therapy and most of the evaluable patients had a demonstrable amount of minimal residual disease after transplantation, as did most of the evaluable stem cell products [16].

Compared with Nordic MCL1 trial, Nordic MCL2 was added with the intensification of the induction therapy to include high-dose cytarabine (3 g/m^2^ every 12 h for a total of 4 doses; patients older than 60 years, cytarabine 2 g/m^2^) and rituximab [22]. In this 2nd Nordic MCL trial, 160 patients with previously untreated MCL less than 66 years of age received intensive therapy including R plus maxi-CHOP alternating with R plus HD-Ara-c. Responders received high-dose chemotherapy with BEAM or BEAC followed by salvage with R-in vivo purged ASCT. When compared with a historical control without HD-Ara-c and R [16], the 2nd Nordic MCL trial had significantly higher ORR (96% vs. 76%, *p* < 0.001) and CR rates (54% vs. 27%, *p* < 0.001). After a median follow-up of 11.4 years, the mOS and mPFS were 12.7 years and 8.5 years, respectively. Although half of the patients were still alive and 40% went under first remission after more than 12 years, an excess disease-related mortality was observed. Additionally, 20 cases (12.6%) of secondary malignancies were reported [9].

Compared with Nordic MCL2 trial, Nordic MCL3 trial had similar treatment with the MCL2 trial except for the addition of 90Y-Ibritumomab Tiuxetan **(**Zevalin) before transplant. In the Nordic MCL3 trial [29], 160 untreated MCL patients < 66 years received R-maxi-CHOP alternating with R-high-dose cytarabine (6 cycles total), followed by high-dose BEAM/BEAC and ASCT. Zevalin was given to responders not in CR before transplantation. The outcome did not differ from that of the MCL2 trial: OS, EFS, and PFS at year 4 were 78, 62, and 71%, respectively. For responding non-CR patients receiving Zevalin, the duration of response was shorter than for the CR group. Inferior PFS, EFS, and OS were predicted by positron emission tomography (PET) scan positivity prior to transplant and detectable minimal residual disease (MRD) post-transplant. Thus, intensification with Zevalin may be too late to improve the outcome of patients not in CR before the transplant.

Therefore, the Nordic MCL1 trial indicated an important strategy to improve the outcome of younger MCL patients – to intensify the induction chemotherapy. The Nordic MCL2 trial provided strong evidence of intensive induction chemotherapy including R plus HD-Ara-c for the dramatic improvement of clinical outcome with acceptable toxicities. Additionally, the Nordic MCL3 trial suggested the need for an early intensified induction therapy as the front-line treatment for younger MCL patients.

#### R-DHAP followed by ASCT

Another form of HD-Ara-c in the induction therapy is DHAP (Dexamethasone, HD-Aara-c, Platinum salt). Nevertheless, the efficacy of R-DHAP regimen is not only due to HD AraC but also the platinum salt. A prospective phase III LyMa Trial assessed R-DHAP as the induction therapy for younger MCL patients. In this study, R-DHA-Cisplatin combined with ASCT has been used as the first-line for the treatment of 299 MCL patients (median age: 57y, range: 27–65), with maintenance treatment by either rituximab or the wait and watch strategy. CR/Cru rates were 81.4 and 92% before and after the transplant, respectively. At a median follow-up of 35.8 months, mPFS and the mOS were not reached. The estimated 3y-PFS and OS were 73.7 and 82.6%, respectively [30]. R-DHA-Cisplatin was also used alternating with R-CHOP for induction therapy before transplantation in patients who did not reach at least a PR after 4 courses of R-DHA-Cisplatin. In the MCL Younger Trial (up to the age of 65 years) of the European Mantle Cell Lymphoma (EMCL) study, alternating courses of CHOP (3 cycles) and DHA-Cisplatin (3 cycles) plus R followed by a H-D Ara-C myeloablative regimen and ASCT (group B) increased CR rates and time to treatment failure (TTF) when compared to 6 courses of CHOP plus R followed by myeloablative chemo radiotherapy and ASCT (group A) in MCL. Conditioning regimen of group A was total body irradiation (TBI) + CTX and group B was TAM (TBI + HD-AraC + melphalan). The ORR for two groups before transplantation was similar (90% vs. 94%; *p* = 0.19), and CR rate of group B was significantly higher than group A (26% vs. 39%, *P* = 0.012). This indicated that the CR rate could be significantly improved when DHA-Cisplatin (combination form of HD-Ara-c) was added to induction therapy with R-CHOP [31]. After a median follow-up of 6.1 years [32], Group B had a significantly longer TTF (median 9.1 years [95% CI 6.3–not reached], 5 year rate 65% [95% CI 57–71]) compared to group A (3.9 years [3.2–4.4], 40% [33–46]; hazard ratio 0.56; *p* = 0.038). During induction immunochemotherapy, group B had increased grade 3 or 4 hematological toxicity and grade 1 or 2 renal toxicity.

In another multicenter Phase II study from France Groupe d’Etude desLymphomes de l’Adulte, HD-Ara-c was added to the induction chemotherapy prior to ASCT in 60 cases of young MCL patients (median age 57.5, range 40–66) with advanced stage. Serial induction therapy consisted of 3 courses of CHOP, followed by 3 courses of DHA-Cisplatin regimen. The addition of rituximab was started from the third CHOP regimen. The conditioning regimen TAM was used as the early consolidation prior to ASCT. A total of 93% of patients responded to R-CHOP regimen, but only 12% of patients achieved CR. After R-DHA-Cisplatin chemotherapy, CR rate increased to 57%. Seven patients did not complete the planned treatment, including three due to disease progression and four due to toxicity, which was mainly renal insufficiency. Finally, 49 patients underwent ASCT, while only one failure in stem cell collection was found. After a median follow-up of 5.6 years, the 5-year EFS and OS was 64 and 75%, respectively. No treatment-related deaths and MDS or AML events occurred. Surprisingly, high incidence of other secondary tumors was observed in 11 patients, of which 5 were kidney cancer [33].

In a subgroup analysis of the LyMa Trial, a comparison was made between the three platinum salt in R-DHAP regimesn for young previously untreated MCL patients (*n* = 298) [34]. The results showed that R-DHA-Oxaliplatin is a better induction regimen before ASCT than R-DHA-Carboplatin and R-DHA-Cisplatin in MCL patients. MCL patients treated with R-DHA-Oxaliplatin had longer PFS and OS. Compared with carboplatin and oxaliplatin, Cisplatin showed more toxicity particularly concerning renal failure. All cases of renal failure during induction (*n* = 8) occurred in Cisplatin group. Twenty-seven (15%) and 38 (21%) out of 184 patients in cisplatin group switched to carboplatin and oxaliplatin, respectively.

EBMT/ EMCL had come up with a consensus for ASCT as the standard first-line consolidation therapy in MCL, giving it a big role in regards to intense therapy regimens [28]. ASCT had high response rates and significantly prolonged survival, especially PFS, with acceptable toxicity. In a randomized controlled trial, the European MCL Network compared two groups with or without ASCT for consolidation. The study revealed that the PFS was significantly prolonged in patients aged younger than 65 years with advanced stage, who had consolidation with ASCT compared to the interferon group (IFN) (39 months vs. 17 months, *P* = .0108) [21]. On the other hand, 85% of patients in the ASCT group had infectious complications due to cytopenias, while this was the case for only 22% of the IFN group. The mortality was 5% in the ASCT group, while 0% in the IFN group. In addition, there was no significant difference in OS between the two groups [21]. This may also be explained by the fact that some patients in the IFN group who experienced relapse subsequently received transplantation. A longer follow-up is needed to determine the effect on OS.

EBMT/ EMCL had also reached the consensus that CR or PR must be achieved before ASCT [28]. The vast majority of clinical trials showed that the outcome of SCT in relapsed MCL patient was often poor, with the expected PFS of about 20 to 40% [35]. Therefore, more recent studies evaluated ASCT as a consolidation therapy in patients sensitive to chemotherapy after the first remission. As shown in Table 1, the median PFS was prolonged to greater than 5 years for the MCL patients receiving ASCT in the first remission, with acceptable toxicity [8, 9, 22, 36, 37]. Meanwhile, for young MCL patients who do not achieve CR after induction therapy, ASCT can further improve the CR rate by 20–40% based on the prior treatments according to MCL net data [31].

Thus far, there are no prospective studies on the choice of conditioning regimen. As demonstrated in the large retrospective study SFGM-TC [38], it was difficult to prove which conditioning regimen was optimal. Additionally, different induction therapies prior to preparative treatment increases the difficulty of comparing between preparative regimens. According to the NCCN NHL Database study [26], commonly used conditioning regimens includes BEAM, TAM (TBI-aracytine- melphalan), CBV (cyclophosphamide, BCNU, VP-16) and TBI/Cy. The MCL network Phase III trial showed that alternative R-CHOP/R-DHAP induction followed by TAM had a longer PFS when compared to R-CHOP followed by TBI/Cy [31]. MCL is sensitive to TBI. However, if the conditioning and induction regimen consist of TBI without HD-Aar-c or other aggressive chemotherapy before transplantation, it seems to be difficult to benefit from the OS. Another comparative retrospective analysis of the European MCL (with TBI) and MCL Nordic group (no TBI), which used a similar induction regimen containing HD Ara-C, showed that TBI seems to improve PFS only in the group of patients who are in PR before ASCT [39]. Because the goal of most front-line induction regimens is CR, TBI is no longer commonly used in Europe and instead, the BEAM regimen has become the new standard [40].

### Moderately intensive therapeutic regimens (Table 3)

#### R-EPOCH regimen

A trial conducted by National Cancer Institute, Bethesda enrolled a total of 26 patients with median age of 57 years (range: 22–73 years) with previously untreated MCL who were treated with 6 courses of Dose-adjusted -EPOCH-R (etoposide, prednisone, vincristine, cyclophosphamide, and doxorubicin combined with rituximab) followed by 5 courses of immunotherapy plus autologous tumor-derived idiotype (Id) -vaccine. The study showed a good response rate, including CR rate of 92% and PR rate of 8%. After a median follow-up of 11 years, the mPFS and mOS were 2 years and 8.7 years, respectively [41]. Another phase II clinical trial reported by the same group used Dose-adjusted-EPOCH-R combined with bortezomib and achieved a considerably high CR rate (up to 92%) in patients with newly diagnosed MCL. Forty-three cases, aged 41–75 years, were included in the trial [42]. However, no detailed age related subgroup analysis was conducted for the response or outcome of the treatment in both these studies.

**Table 3:** Moderate intensity of front-line therapy for young fit patient with mantle cell lymphoma

Regimen	Series	No.	Age(ys) Median (range)	MIPI Low/Int/High(%)	ORR (%)	CR (%)	mFU (ms)	mPFS (ms)	mOS (ms)	TRM (%)	Second malignancy(%)
Bort-DA-EPOCH-R ± Bort	Dunleavy (2012) [42]	43	58(41–73)	50/37/13	92	63	48	50% (4-y PFS)	80% (4-y OS)	na	na
DA-EPOCH-R + vaccine	Grant (2011) [41, 67]	26	57 (22–73)	65/16/19	100	92	11ys	24	104	na	na
RB/RC	Armand(2016) [43]	23	57 (42–69)	70/22/9	96	96	13	96% (1-y PFS)	96% (1-y OS)	0	na
*R* ^2^	Ruan (2015) [60]	38	65(42–86)	34/34/32	92	64	30	85% (2-y PFS)	97% (2-y OS)	0	23.3
R-CHOP+ Zevalin	Smith(2007) [45, 46]	56	61 (33–83)	50/27/12	74	42	9.8ys	na	56% at 10 ys (age ≤ 65)	0	10.7

Abbreviations as indicated in the Tables 1 and 2

Note: *Bort* Bortezomib, *DA-EPOCH* dose-adjusted etoposide, doxorubicin, and cyclophosphamide with vincristine, prednisone, *RB/RC* rituximab/bendamustine+ rituximab/high-dose cytarabine; *R*^2^ rituximab plus lenalidomide;

#### RB/R-Arac

A phase II single-arm clinical trial from two centers (Dana-Farber Cancer Institute and Beth Israel Deaconess Medical Center) tested the induction regimen of RB (rituximab/bendamustine) followed by RC (rituximab/high-dose cytarabine) for 23 cases (median age: 57, range, 42–69), who were transplant-eligible with newly diagnosed MCL. Patients received three cycles of RB followed by three cycles of RC. This induction regimen had a considerable high CR/unconfirmed rate (96%) and MRD-negative rate (93% in 15 evaluable patients). Twenty-one patients received consolidation with ASCT. After a median follow-up of 13 months, the median PFS was found to be 96%. No TRM was observed. However, the high remission rates found in this study may relate to a higher proportion of low-risk patients (70%). In addition, the follow-up time for the study was short, which warrants further observation [43].

#### CdM (cladribine (2-CdA) and mitoxantrone)

CdM was used for the treatment of 62 cases of MCL and low grade NHL with a median age of 59 (31–76) years. CdM displayed high activity for MCL with an ORR of 100%. However, the CR rate was only 44%. In 9 patients with previously untreated MCL, the ORR also reached 100%, while the CR rate was found to be 33%. Among all patients, myelosuppression was the major toxicity with 23% grade 3 and 50% grade 4 granulocytopenia [44]. However, the results of this study are controversial since the study included only a few patients with previously untreated MCL.

#### R-CHOP+ Zevalin

A phase II trial of R-CHOP followed by Zevalin in untreated MCL was performed by the Eastern Cooperative Oncology Group Study E1499 [45]. Fifty-six patients with 91% of stage III/IV and 78% of marrow-positive were enrolled between November 2003 and February 2005. Median age was 61 (range: 33–83) years while low to intermediate MIPI score patients accounted for 77%. After four cycles of R-CHOP, responding (CR/PR) and stable patients received Zevalin. Fifty-one (91%) patients received all treatment, with 42% CR/Cru, 32% PR, 12% stable and 4% unevaluable, and an improvement in response in 16 patients after Zevalin. At a median follow-up of 9.8 years, median OS for those patients under 65 years has not been reached at 10 years. No therapy related myeloid neoplasia was observed in this trial. The second malignancies include non-small cell lung cancer (*N* = 2), bladder cancer (*N* = 1), ampullary adenocarcinoma (*N* = 1) and resected localized non-melanoma skin cancers (*N* = 2) [46].

### Less intensive therapeutic regimens (Table 4)

#### R-CHOP

Howard, O. M. et al. conducted a phase II study with six cycles of R-CHOP regimen as the induction therapy in 40 patients (median age: 55 years, range: 31–69) with newly diagnosed MCL. The addition of monoclonal anti-CD20 antibody R to CHOP regimen significantly improved the ORR to 96% and CR / unconfirmed CR (CRu) rate to 48% [20], which were in accordance with the results from a prospective randomized trial of the German Low Grade Lymphoma Study Group (ORR: 94% in RCHOP vs 75% in CHOP, *P* = 0.0054; CR: 34% in RCHOP vs 7% in CHOP; *P* = 0.00024) [47]. However, the duration of remission was short, with a median PFS time of only 16.5 months at a median follow-up of 25 months [19, 20]. Also, no differences were observed for PFS in the two studies [20, 47]. Compared with CHOP regimen, R-CHOP could not translate favorable clinical and molecular response rates into prolonged PFS. Nevertheless, R-CHOP may transiently clear peripheral-blood (PB) or bone marrow (BM) of detectable tumor cells [20]. Another study by LaCasce and colleagues [26] showed poor survival advantage for the newly diagnosed younger MCL patients receiving R-CHOP alone. R-CHOP (*n* = 29) demonstrated inferior PFS compared with other aggressive regimens (*n* = 138) (*P* < .004).

**Table 4:** Less intensive front-line therapy for young fit patient with mantle cell lymphoma

Regimen	Series	No.	Age (ys) Median (range)	MIPI Low/Int/High (%)	ORR (%)	CR (%)	mFU (ms)	mPFS (ms)	mOS (ms)	TRM (%)	Second malignancy(%)
R-CHOP	Howard (2002) [20]	40	55 (31–69)	na	96	48	25	16.5	na	2.5	na
R-CHOP	LaCasce (2012) [26]	29	55 (< 65)	21/76/3 (IPI)	na	na	33	18% 3-y PFS	69% 3-y OS	31	na
R-CHOP Vs. CHOP	Lenz (2005) [47]	38	< 65	28/66/7 ^a^ (IPI)	94^a^	34^a^	18	NR^a^	NR^a^	2^a^	na
		39	< 65	20/70/10^a^ (IPI)	75^a^	7^a^	18	NR^a^	NR^a^	0^a^	na
R-CHOP Vs. VR-CAP	Robak(2015) [50]	244	66(34–82)	29/38/33	89	42	40	16.1	56.3	6	na
		243	65(26–88)	31/40/29	92	53	40	30.7	NR	5	na
B-R + Ibrutinib	Maddocks(2015) [48]	17 5 (untreated)	na (62–72)	na	94	76	na	NR	NR	na	na
R-CHOP+ Ibrutinib	Younes (2014) [49]	5	na	na	94 (all subtypes)	72	7.1	na	na	na	na

Abbreviations as indicated in the Table 1

Note: *VR-CAP* rituximab, cyclophosphamide, doxorubicin, bortezomib, prednisone, *B-R* bendamustine and rituximab

^a^ including younger than 65 years and older than 65 years

#### B-R + Ibrutinib

A phase I/Ib study from 2012 to 2014 conducted in Ohio State University demonstrated that ibrutinib in combination with bendamustine and rituximab in 48 patients with B-cell lymphoma, which included 17 patients with MCL (5 untreated, ages 62–72), produced an ORR and CR rate for MCL of 94 and 76%, respectively. The mPFS and mOS that were not reached along with an acceptable toxicity profile. However, the sample size of untreated young patients with MCL in this study was too small to make any significant conclusions [48].

#### R-CHOP+ Ibrutinib

In a phase 1b, open-label, non-randomized study from 2012 to 2013 at six centers in the USA and France involving 32 patients (ages 18 years or older) with CD20 positive B-cell NHL, five patients with MCL received ibrutinib combined with standard doses of R-CHOP regimen. While the ORR and CR rate for all patients was 94 and 72%, respectively, and the combination was found to be safe, the sample size of MCL was small to make any significant conclusions [49]. Larger sample sizes are needed to test this regimen in phase III trials.

#### VR-cap

A pivotal phase III trial called the LYM-3002 study from 2008 to 2011 was conducted at 128 sites in 28 countries across Europe, Asia, North America, and South America. This study compared 244 patients who received R-CHOP with 243 patients who received VR-CAP (vincristine in R-CHOP regimen was replaced by bortezomib) and all were previously untreated with MCL. Approximately one third of patients were less than 60 years of age. CR rate in the VR-CAP group was 53%, which was slightly higher than 42% of R-CHOP group. After median follow-up of 40 months, median duration of CR in VR-CAP group was significantly longer (42.1 months vs. 18.0 months). Meanwhile, the PFS was increased by 96%, which almost doubled (16.1 months vs. 30.7 months, *p* < 0.001). There was no significant difference in mOS between the two groups. However, outcome analysis according to age subgroups was lacking [50].

### The significance of MRD before and after ASCT for MCL patients treated with front-line therapy

The depth of remission before and after ASCT can markedly affect the outcome of MCL patients. Even at CR prior or post ASCT, the depth of remission was crucial for long term survival. The presence of a clonal IgH rearrangement or t (11; 14) by PCR or positive flow cytometry from blood/bone marrow was considered positive for MRD. MRD positivity prior to transplantation was associated with shorter OS and PFS, although no analysis was made according to age subgroup [51].

An increase of ORR and CR is a result of the addition of R to induction regimen, aiding in the improvement of PFS [52]. This indicates the importance of eradicating residue lymphoma cells. In the 2 randomized trials of the European MCL Network, multivariate analysis showed that the MRD status before ASCT or maintenance is one of the strongest independent prognostic factors [53]. In addition, in the Nordic MCL3 trial, positive PET pre-transplant and detectable MRD post-transplant predicted inferior PFS, EFS, and OS [29]. A phase III, randomized, controlled and multicenter study also showed a commendable high level of molecular remission rate (72 and 53% by nested PCR, as well as 80 and 67% by RQ-PCR before ASCT in PB and BM, respectively) had been translated into a 2-year PFS and OS rates of 77 and 88%, respectively, for intermediate to high-risk MIPI score patients with MCL [54]. Furthermore, the LyMa trial demonstrates for the first time that RM after ASCT prolongs EFS, PFS, and OS [40].

Recently, Next-Generation Sequencing (NGS) has been emerged as a tool to quantify the MRD in B-cell lymphoma [55–58]. Meanwhile, the circulating tumor DNA (ctDNA) for tumor-specific rearrangements of the immunoglobulin receptor (VDJ) gene sequences was utilized as MRD detection in B-cell lymphoma [57–59]. The studies showed that the kinetics of ctDNA clearance was predictive of a clinical outcome. Furthermore, low molecular level of ctDNA can be detected by NGS with great sensitivity and specificity. NGS can identify genome-wide tumor-derived alterations in ctDNA. Thus monitoring ctDNA in the peripheral blood by NGS could be a tool assisting in both the selection of patients for maintenance or pre-emptive treatment.

### Novel agents and ongoing trials on front-line therapy for young MCL patients (Table 5)

Due to acute and long term toxicity of conventional chemotherapy regimen and drug resistance, more and more novel agents have been included in the front-line therapy in clinical trials. The combination of rituximab and lenalidomide (R^2^) was tested as an initial treatment for MCL (median age: 65 years) in a small single-group, multicenter, phase II study. However, the patients enrolled were not fit for transplantation because of co-existing conditions or wished to avoid combination intensive chemotherapy. This combination therapy showed considerable activity with ORR of 92% and CR rate of 64% [60]. A phase III trial “Triangle” is assessing whether the implementation of a BTK-inhibitor in first-line treatment may be able to replace ASCT consolidation for younger MCL patients [61]. A phase II window trial for young untreated MCL patients is currently ongoing at MDACC, which includes a two-part window protocol: ibrutinib and R followed by RHyper-CVAD alternating with R-MA for fewer cycles, if the patient demonstrates a good response with ibrutinib. The preliminary results shows an ORR of 100%, which is more than promising [62]. Therefore, “chemo-free” and novel agents incorporated to conventional chemotherapy regimens can be the promising front-line therapy for MCL patients.

**Table 5:** Ongoing trials using novel agents alone or in combination as front-line therapy for young MCL patients

Trial	Regimen	Design	Sponsor	ClinicalTrials.gov Identifier
Window	IR with With Hyper-CVAD Consolidation	Phase II, Open-Label	M.D. Anderson Cancer Center	NCT02427620
–	O-HyperCVAD/ O-MA	Phase II, Open Label	Roswell Park Cancer Institute	NCT01527149
Triangle	(1) R-CHOP /R-DHAP + ASCT (2) R-CHOP+IBN/R-DHAP +ASCT +IBN maintenance ×2 ys (3) R-CHOP+IBN/ R-DHAP + IBN maintenance × 2 ys	Phase III, Open-Label	Prof. Dr. M. Dreyling	NCT02858258
ECOG-ACRIN (EA4151)	patients with MRD (−) after induction: (1)ASCT + R maintenance Open-Label (2) R maintenance without ASCT	Randomized Phase III, Open-Label	ECOG-ACRIN Cancer Research Group	NCT03267433
BDH-MCL01	R-EDOCH/R-DHAP→HDT/ASCT or R-EDOCH/R-DHAP→MR or MTp	Phase III, Open-Label	Institute of Hematology & Blood Diseases Hospital	NCT02858804
–	IBN^a^	Phase II, Open Label	M.D. Anderson Cancer Center	NCT03282396
–	BTZ	retrospective and prospective	Xian-Janssen Pharmaceutical Ltd.	NCT03053024
–	VCR	Phase II, Open-Label	University of Arizona	NCT00980395
–	BR/RAC	Phase I, Open-Label	Washington University School of Medicine	NCT 02728531
–	R-CHOP14 → R-HIDAC→RIT → HDT → ASCT	Phase I/II, Open-Label	Memorial Sloan Kettering Cancer Center	NCT 01484093

Abbreviations as indicated in the Tables 1, 3 and 4

Note: *IR* ibrutinib and rituximab, *O-HyperCVAD/ O-MA* Hyper-Fractionated Cyclophosphamide, Doxorubicin, Vincristine and Dexamethasone Alternating With Ofatumumab High-Dose Cytarabine and Methotrexate, *R-EDOCH* rituximab etoposide, dexamethasone, doxorubicin, cyclophosphamide, and vincristine, *HDT* high-dose chemotherapy, *MR* maintenance rituximab, *MTp* maintenance thalidomide and prednisone, *BTZ* Bortezomib, *RCT* Rituximab, Cladribine, and Temsirolimus, *VCR* Bortezomib (Velcade), Cladribine and Rituximab, *RB* Rituximab/Bendamustine, *RAC* Rituximab/Cytarabine, *R-CHOP-14* rituximab, cyclophosphamide, doxorubicin, vincristine, and prednisone every 2 weeks, *RIT* radioimmunotherapy Iodine ^131^I Tositumomab

^a^for the low-risk disease

## Conclusion

For young and fit newly diagnosed MCL patients, a front-line therapy is extremely crucial. As demonstrated by studies utilizing intensive therapies, the longest duration of response and survival up to 13.4 years can be obtained. However, this is also accompanied by long-term side effects. So far, the standard of therapy includes induction with R combined with a series of aggressive chemotherapy consisting of HD-Ara-c, followed by ASCT consolidation in first remission. The benefits of R maintenance has also been demonstrated. Furthermore, MRD negativity will be the optimal therapeutic goal to achieve before and after ASCT. Some novel therapeutic strategies appear to improve the outcome, but it is not yet entirely clear how these results translate into the large size population. Of note, MCL patients need to be stratified at diagnosis and be provided with different intensities of front-line regimen. A risk-stratified approach should be evaluated for this heterogeneous population. In the future, based on the identification of specific biomarker, a novel agent can be used as a single agent or combined with conventional chemotherapy regiments, in hopes of reducing toxicity. A more sensitive molecular monitoring, such as ctDNA, could be a tool assisting in both the selection of patients for maintenance or pre-emptive treatment.

## Abbreviations

AML: Acute myeloid leukemia
ASCT: Autologous stem cell transplantation
BEAC: Carmustine, etoposide, cytarabine, and cyclophosphamide
BEAM: Carmustine etoposide, cytarabine, and melphalan
BM: Bone marrow
BR: Bendamustine plus rituximab
CBV: Cyclophosphamide, BCNU, VP-16
CR: Complete response rate
ctDNA: Circulating tumor DNA
EMBT: European Group for Blood and Marrow Transplantation
EMCL: European Mantle Cell Lymphoma Network
HD-Ara-c: High-dose cytarabine
HDT/ASCR: High dose chemotherapy/ autologous stem cell rescue
MCL: Mantle cell lymphoma
MDS: Myelodysplastic syndrome
MIPI: Mantle cell lymphoma-International Prognostic Index
mOS: Median overall survival
NGS: Next-Generation Sequencing
ORR: Overall response rate
PB: Peripheral-blood
PFS: Progression-free survival
R^2^: Rituximab and lenalidomide
RB: Rituximab/bendamustine
RC: Rituximab/high-dose cytarabine
R-CHOP: Rituximab, cyclophosphamide, doxorubicin, vincristine and prednisone
R-Hyper-CVAD: Fractionated cyclophosphamide, vincristine, doxorubicin, and dexamethasone
TAM: TBI-aracytine- melphalan
TBI: Total body irradiation
TRM: Treatment-related mortality

## References

[1] Morton LM, Wang SS, Devesa SS, Hartge P, Weisenburger DD, Linet MS (2006). Lymphoma incidence patterns by WHO subtype in the United States, 1992-2001. Blood.

[2] Sant M, Allemani C, Tereanu C (2010). Incidence of hematologic malignancies in Europe by morphologic subtype: results of the HAEMACARE project. Blood.

[3] Zhou Y, Wang H, Fang W (2008). Incidence trends of mantle cell lymphoma in the United States between 1992 and 2004. Cancer.

[4] Aschebrook-Kilfoy B, Caces DB, Ollberding NJ, Smith SM, Chiu BC (2013). An upward trend in the age-specific incidence patterns for mantle cell lymphoma in the USA. Leuk Lymphoma.

[5] Dreyling M, Hiddemann W, European MCLN. Current treatment standards and emerging strategies in mantle cell lymphoma. Hematol Educ Program Am Soc Hematol Am Soc Hematol Educ Program. 2009. p. 542–51. 10.1182/asheducation-2009.1.542 20008239

[6] Kluin-Nelemans HC, Hoster E, Hermine O (2012). Treatment of older patients with mantle-cell lymphoma. N Engl J Med.

[7] Chihara D, Cheah CY, Westin JR (2016). Rituximab plus hyper-CVAD alternating with MTX/Ara-C in patients with newly diagnosed mantle cell lymphoma: 15-year follow-up of a phase II study from the MD Anderson Cancer Center. Br J Haematol.

[8] Geisler CH, Kolstad A, Laurell A (2012). Nordic MCL2 trial update: six-year follow-up after intensive immunochemotherapy for untreated mantle cell lymphoma followed by BEAM or BEAC + autologous stem-cell support: still very long survival but late relapses do occur. Br J Haematol.

[9] Eskelund CW, Kolstad A, Jerkeman M, et al. 15-year follow-up of the second Nordic mantle cell lymphoma trial (MCL2): prolonged remissions without survival plateau. Br J Haematol. 2016;175(3):410–8 10.1111/bjh.14241 27378674

[10] Stewart DA, Vose JM, Weisenburger DD (1995). The role of high-dose therapy and autologous hematopoietic stem cell transplantation for mantle cell lymphoma. Ann Oncol.

[11] Ketterer N, Salles G, Espinouse D (1997). Intensive therapy with peripheral stem cell transplantation in 16 patients with mantle cell lymphoma. Ann Oncol.

[12] Haas R, Brittinger G, Meusers P (1996). Myeloablative therapy with blood stem cell transplantation is effective in mantle cell lymphoma. Leukemia.

[13] Khouri IF, Romaguera J, Kantarjian H (1998). Hyper-CVAD and high-dose methotrexate/cytarabine followed by stem-cell transplantation: an active regimen for aggressive mantle-cell lymphoma. J Clin Oncol.

[14] Milpied N, Gaillard F, Moreau P (1998). High-dose therapy with stem cell transplantation for mantle cell lymphoma: results and prognostic factors, a single center experience. Bone Marrow Transplant.

[15] Freedman AS, Neuberg D, Gribben JG (1998). High-dose chemoradiotherapy and anti-B-cell monoclonal antibody-purged autologous bone marrow transplantation in mantle-cell lymphoma: no evidence for long-term remission. J Clin Oncol.

[16] Andersen NS, Pedersen L, Elonen E (2003). Primary treatment with autologous stem cell transplantation in mantle cell lymphoma: outcome related to remission pretransplant. Eur J Haematol.

[17] Vandenberghe E, Ruiz de Elvira C, Loberiza FR (2003). Outcome of autologous transplantation for mantle cell lymphoma: a study by the European blood and bone marrow transplant and autologous blood and marrow transplant registries. Br J Haematol.

[18] Mangel J, Leitch HA, Connors JM (2004). Intensive chemotherapy and autologous stem-cell transplantation plus rituximab is superior to conventional chemotherapy for newly diagnosed advanced stage mantle-cell lymphoma: a matched pair analysis. Ann Oncol.

[19] Foran JM, Rohatiner AZ, Cunningham D (2000). European phase II study of rituximab (chimeric anti-CD20 monoclonal antibody) for patients with newly diagnosed mantle-cell lymphoma and previously treated mantle-cell lymphoma, immunocytoma, and small B-cell lymphocytic lymphoma. J Clin Oncol.

[20] Howard OM, Gribben JG, Neuberg DS (2002). Rituximab and CHOP induction therapy for newly diagnosed mantle-cell lymphoma: molecular complete responses are not predictive of progression-free survival. J Clin Oncol.

[21] Dreyling M, Lenz G, Hoster E (2005). Early consolidation by myeloablative radiochemotherapy followed by autologous stem cell transplantation in first remission significantly prolongs progression-free survival in mantle-cell lymphoma: results of a prospective randomized trial of the European MCL network. Blood.

[22] Geisler CH, Kolstad A, Laurell A (2008). Long-term progression-free survival of mantle cell lymphoma after intensive front-line immunochemotherapy with in vivo-purged stem cell rescue: a nonrandomized phase 2 multicenter study by the Nordic lymphoma group. Blood.

[23] Hermine OR, Hoster E, Walewski J (2011). Alternating courses of 3x Chop and 3x Dhap plus rituximab followed by a high dose Ara-C containing Myeloablative regimen and autologous stem cell transplantation (Asct) is superior to 6 courses of Chop plus rituximab followed by Myeloablative Radiochemotherapy and Asct in mantle cell lymphoma: update of results of the mcl younger trial of the European mantle cell lymphoma network (mcl net). Ann Oncol.

[24] Merli F, Luminari S, Ilariucci F (2012). Rituximab plus HyperCVAD alternating with high dose cytarabine and methotrexate for the initial treatment of patients with mantle cell lymphoma, a multicentre trial from Gruppo Italiano studio Linfomi. Br J Haematol.

[25] Bernstein SH, Epner E, Unger JM (2013). A phase II multicenter trial of hyperCVAD MTX/Ara-C and rituximab in patients with previously untreated mantle cell lymphoma; SWOG 0213. Ann Oncol.

[26] LaCasce AS, Vandergrift JL, Rodriguez MA (2012). Comparative outcome of initial therapy for younger patients with mantle cell lymphoma: an analysis from the NCCN NHL database. Blood.

[27] Romaguera JE, Fayad L, Rodriguez MA (2005). High rate of durable remissions after treatment of newly diagnosed aggressive mantle-cell lymphoma with rituximab plus hyper-CVAD alternating with rituximab plus high-dose methotrexate and cytarabine. J Clin Oncol.

[28] Robinson S, Dreger P, Caballero D (2015). The EBMT/EMCL consensus project on the role of autologous and allogeneic stem cell transplantation in mantle cell lymphoma. Leukemia.

[29] Kolstad A, Laurell A, Jerkeman M (2014). Nordic MCL3 study: 90Y-ibritumomab-tiuxetan added to BEAM/C in non-CR patients before transplant in mantle cell lymphoma. Blood.

[30] Steven Le Gouill CT, Lucie Oberic, Krimo Bouabdallah, Emmanuel Gyan, Gandhi Damaj, Vincent Ribrag, Serge Bologna, Remy Gressin, Olivier Casasnovas, Corinne Haioun, Philippe Solal-Celigny, Herve Maisonneuve, Eric Van Den Neste, Anne Moreau, Marie C Bene, Gilles Salles, Herv Tilly, Thierry Lamy, and Olivier Hermine: Rituximab maintenance versus wait and watch after four courses of R-DHAP followed by autologous stem cell transplantation in previously untreated young patients with mantle cell lymphoma: first interim analysis of the phase III prospective Lyma trial, a Lysa Study. Blood 2014(21):124.

[31] Olivier Hermine EH, Walewski J, Ribrag V, Brousse N, Thieblemont C, Bouabdallah R, Dohner H, Feugier P, Forspointner R, Haioun C, Kneba M, Hänel M, Casasnovas O, Mertelsmann RH, Hallek M, Andre Bosly MN, Klapper W, Gisselbrecht C, Coiffier B, Unterhalt M, Hiddemann W, Dreyling MH. Alternating courses of 3x CHOP and 3x DHAP plus rituximab followed by a high dose ARA-C containing Myeloablative regimen and autologous stem cell transplantation (ASCT) increases overall survival when compared to 6 courses of CHOP plus rituximab followed by Myeloablative Radiochemotherapy and ASCT in mantle cell lymphoma: final analysis of the MCL younger trial of the European mantle cell lymphoma network (MCL net). Blood. 2012;120(21)

[32] Hermine O, Hoster E, Walewski J (2016). Addition of high-dose cytarabine to immunochemotherapy before autologous stem-cell transplantation in patients aged 65 years or younger with mantle cell lymphoma (MCL younger): a randomised, open-label, phase 3 trial of the European mantle cell lymphoma network. Lancet.

[33] Delarue R, Haioun C, Ribrag V (2013). CHOP and DHAP plus rituximab followed by autologous stem cell transplantation in mantle cell lymphoma: a phase 2 study from the Groupe d'Etude des Lymphomes de l'Adulte. Blood.

[34] Geisler CH, Kolstad A, Laurell A (2010). The mantle cell lymphoma international prognostic index (MIPI) is superior to the international prognostic index (IPI) in predicting survival following intensive first-line immunochemotherapy and autologous stem cell transplantation (ASCT). Blood.

[35] Vose JM (2012). Autotransplantation for mantle cell lymphoma. Cancer J.

[36] Dreger P, Rieger M, Seyfarth B (2007). Rituximab-augmented myeloablation for first-line autologous stem cell transplantation for mantle cell lymphoma: effects on molecular response and clinical outcome. Haematologica.

[37] de Guibert S, Jaccard A, Bernard M, Turlure P, Bordessoule D, Lamy T (2006). Rituximab and DHAP followed by intensive therapy with autologous stem-cell transplantation as first-line therapy for mantle cell lymphoma. Haematologica.

[38] Touzeau C, Leux C, Bouabdallah R (2014). Autologous stem cell transplantation in mantle cell lymphoma: a report from the SFGM-TC. Ann Hematol.

[39] Rubio MT, Boumendil A, Luan JJ (2010). Is there still a place for Total body irradiation (TBI) in the conditioning regimen of autologous stem cell transplantation in mantle cell lymphoma ?: a retrospective study from the lymphoma working party of the EBMT. Blood.

[40] Le Gouill S, Thieblemont C, Oberic L (2016). Rituximab maintenance after autologous stem cell transplantation prolongs survival in younger patients with mantle cell lymphoma: final results of the randomized phase 3 LyMa trial of the Lysa/Goelams group. Blood.

[41] Grant C, Neelapu SS, Kwak LW, Dunleavy K, White T, Miller BW, Jaffe ES, Steinberg SM, Bird BH, Wilson WH (2011). Eleven-year follow-up of Idiotype vaccine and DA-EPOCH-rituximab in untreated mantle cell lymphoma: correlation of survival with Idiotype immune response. Blood.

[42] Dunleavy K, Grant, C., Hessler, J., Miller, B.W., Steinberg, S.M., Pittaluga, S., Roschewski, M., Jaffe, E.S., Wiestner, A. & Wilson, W.H.: Bortezomib plus DA-EPOCH-R induction therapy followed by maintenance bortezomib versus observation in newly diagnosed mantle cell lymphoma. Blood 2012(120): 3672.

[43] Armand P, Redd R, Bsat J (2016). A phase 2 study of rituximab-Bendamustine and rituximab-Cytarabine for transplant-eligible patients with mantle cell lymphoma. Br J Haematol.

[44] Rummel MJ, Chow KU, Karakas T (2002). Reduced-dose cladribine (2-CdA) plus mitoxantrone is effective in the treatment of mantle-cell and low-grade non-Hodgkin's lymphoma. Eur J Cancer.

[45] Smith MR, Zhang L, Gordon LI (2007). Phase II study of R-CHOP followed by 90Y-Ibritumomab Tiuxetan in untreated mantle cell lymphoma: eastern cooperative oncology group study E1499. Blood.

[46] Smith MR, Hong F, Li H (2017). Mantle cell lymphoma initial therapy with abbreviated R-CHOP followed by 90Y-ibritumomab tiuxetan: 10-year follow-up of the phase 2 ECOG-ACRIN study E1499. Leukemia.

[47] Lenz G, Dreyling M, Hoster E (2005). Immunochemotherapy with rituximab and cyclophosphamide, doxorubicin, vincristine, and prednisone significantly improves response and time to treatment failure, but not long-term outcome in patients with previously untreated mantle cell lymphoma: results of a prospective randomized trial of the German low grade lymphoma study group (GLSG). J Clin Oncol.

[48] Maddocks K, Christian B, Jaglowski S (2015). A phase 1/1b study of rituximab, bendamustine, and ibrutinib in patients with untreated and relapsed/refractory non-Hodgkin lymphoma. Blood.

[49] Younes A, Thieblemont C, Morschhauser F (2014). Combination of ibrutinib with rituximab, cyclophosphamide, doxorubicin, vincristine, and prednisone (R-CHOP) for treatment-naive patients with CD20-positive B-cell non-Hodgkin lymphoma: a non-randomised, phase 1b study. Lancet Oncol.

[50] Robak T, Huang H, Jin J (2015). Bortezomib-based therapy for newly diagnosed mantle-cell lymphoma. N Engl J Med.

[51] Cowan AJ, Stevenson PA, Cassaday RD (2016). Pretransplantation minimal residual disease predicts survival in patients with mantle cell lymphoma undergoing autologous stem cell transplantation in complete remission. Biol Blood Marrow Transplant.

[52] Schulz H, Bohlius JF, Trelle S (2007). Immunochemotherapy with rituximab and overall survival in patients with indolent or mantle cell lymphoma: a systematic review and meta-analysis. J Natl Cancer Inst.

[53] S.M. Aukema, E. Hoster, A. Rosenwald*,* et al.: P53 but not SOX11 IHC has prognostic value independent of MIPI AND KI-67 in prospective trials of the european-MCL network. In: 14th International Conference on Malignant Lymphoma Palazzo dei Congressi: 2017; Lugano, Switzerland; 2017.

[54] Sergio Cortelazzo MM, Ladetto M, Ferrero S, Ciccone G, Evangelista A, Mian M, Di Rocco A, Chiappella A, Rossi G, Re A, Zinzani PL, Balzarotti M, Cavallo F, Rusconi C, Gotti M, Arcaini L, Gobbi M, Gomes M, Molinari A, Liberati AM, Michieli M, Latte G, Cabras MG, Novero D, Paulli M, Zamò A, Chilosi M, Federico M, Vitolo U (2015). High dose sequential chemotherapy with rituximab and ASCT as first line therapy in adult MCL patients: clinical and molecular response of the MCL0208 trial, a FIL study. Haematologica.

[55] Komajda M (1996). Genetic factors in familial hypertrophic cardiomyopathy: does molecular cardiology offer new perspectives?. Heart.

[56] Jares P, Colomer D, Campo E (2007). Genetic and molecular pathogenesis of mantle cell lymphoma: perspectives for new targeted therapeutics. Nat Rev Cancer.

[57] Koffi M, Solano P, Barnabe C (2007). Genetic characterisation of Trypanosoma brucei s.L. using microsatellite typing: new perspectives for the molecular epidemiology of human African trypanosomiasis. Infect Genet Evol.

[58] Mechtersheimer G, Lehnert T, Penzel R, Joos S, Egerer G, Otto HF (2003). Gastrointestinal stromal tumors. A morphologic and molecular genetic independent tumor entity with new therapeutic perspectives. Pathologe.

[59] Melani C, Roschewski M (2016). Molecular monitoring of cell-free circulating tumor DNA in non-Hodgkin lymphoma. Oncology (Williston Park).

[60] Ruan J, Martin P, Shah B (2015). Lenalidomide plus rituximab as initial treatment for mantle-cell lymphoma. N Engl J Med.

[61] Dreyling M, Ferrero S, European mantle cell lymphoma N (2016). The role of targeted treatment in mantle cell lymphoma: is transplant dead or alive?. Haematologica.

[62] Wang M, Lee HJ, Thirumurthi S (2016). Chemotherapy-free induction with Ibrutinib-rituximab followed by shortened cycles of chemo-immunotherapy consolidation in young, newly diagnosed mantle cell lymphoma patients: a phase II clinical trial. Blood.

[63] Damon LE, Johnson JL, Niedzwiecki D (2009). Immunochemotherapy and autologous stem-cell transplantation for untreated patients with mantle-cell lymphoma: CALGB 59909. J Clin Oncol.

[64] van 't Veer MB, de Jong D, MacKenzie M (2009). High-dose Ara-C and beam with autograft rescue in R-CHOP responsive mantle cell lymphoma patients. Br J Haematol.

[65] Le Gouill S, Thieblemont C, Oberic L, et al. Rituximab maintenance versus wait and watch after four courses of R-DHAP followed by autologous stem cell transplantation in previously untreated young patients with mantle cell lymphoma: first interim analysis of the phase III prospective Lyma trial, a Lysa Study. Blood. 2014;124(21)

[66] Schaffel R, Hedvat CV, Teruya-Feldstein J (2010). Prognostic impact of proliferative index determined by quantitative image analysis and the international prognostic index in patients with mantle cell lymphoma. Ann Oncol.

[67] Neelapu SS, Kwak LW, Kobrin CB (2005). Vaccine-induced tumor-specific immunity despite severe B-cell depletion in mantle cell lymphoma. Nat Med.

